# Effectiveness and Selectiveness of Traps and Baits for Catching the Invasive Hornet *Vespa velutina*

**DOI:** 10.3390/insects11100706

**Published:** 2020-10-16

**Authors:** Simone Lioy, Daniela Laurino, Michela Capello, Andrea Romano, Aulo Manino, Marco Porporato

**Affiliations:** Department of Agricultural, Forest and Food Sciences, University of Turin, Largo Paolo Braccini 2, 10095 Grugliasco (Turin), Italy; daniela.laurino@unito.it (D.L.); mc.michelacappe@gmail.com (M.C.); rmnndr83@gmail.com (A.R.); aulo.manino@unito.it (A.M.); marco.porporato@unito.it (M.P.)

**Keywords:** Asian yellow-legged hornet, *Vespa velutina*, invasive species, monitoring, surveillance, early detection, wasps, trapping, baits

## Abstract

**Simple Summary:**

The introduction of invasive species is one of the major causes of biodiversity loss and, in many cases, entails considerable economic consequences. Due to their biological traits, social Vespidae are known for their potential in establishing viable populations and become invasive in many countries of the world. A good example is represented by the invasion success of the Asian yellow-legged hornet *Vespa velutina*, a species that has been introduced and established in Europe and other countries of Asia. By preying on honey bees and native insects, this species is a threat for the biodiversity, the pollination ecosystem service and the economy. Traps for wasps and hornets are widely used for monitoring the presence of *V. velutina* or, at a rather higher density, as a complementary method for limiting its impacts. Here we compared the performance of two typologies of traps and baits widely used for trapping this invasive species and, at the same time, evaluated the consequences of this activity on native insects. Findings highlighted that the performance of the trap/bite combinations changed in relation to the season. However, the proportion of non-target insects in the traps stress the necessity of developing alternative monitoring and control techniques.

**Abstract:**

*Vespa velutina* is an invasive hornet that is colonising several countries worldwide, with detrimental effects on multiple components but primarily affecting honey bees and native insect species. Traps for wasps and hornets are commonly used for trapping *V. velutina*, both for monitoring and control purposes. In this study, we compared the performances of two typologies of traps and baits widely used for trapping this invasive hornet, by evaluating their effectiveness and selectiveness in trapping *V. velutina* in two sites during two different periods of the year, spring and autumn. The performance of the traps changed in relation to (i) the trap’s model, (ii) the bait’s typology and (iii) the period of the year. In spring, traps with common beer as bait were more effective and more selective independently of trap’s model than the commercial bait that has been tested. On the contrary, in autumn, just one combination of trap and attractant (the commercial trap and bait) achieved higher effectiveness and selectiveness. Despite the underlined variations among traps and baits, overall catches of *V. velutina* were scanty compared to bycatches of non-target insects, since best performing traps either in term of effectiveness and selectiveness caught 3.65% of the target species in spring and 1.35% in autumn upon the total trapped insects. This highlights the urgent necessity of developing more selective trapping methods for monitoring and particularly for controlling purposes.

## 1. Introduction

The Asian yellow-legged hornet *Vespa velutina* is an invasive species, introduced in Europe [1] and in a few other non-native countries of Asia [2,3], where it has established viable and expanding populations [4]. As in other hornet and wasp species, the larval stages of *V. velutina* require proteins for their development, which are commonly obtained by preying on other insects such as bees, other wasps or flies, but with a preference for honey bees [5,6]. In the invaded countries of Europe, the intensive predation of this hornet towards *Apis mellifera* could lead to serious impacts on honey bee colonies, due to the induced foraging paralysis, the homing failure of foraging bees [7] and the absence of an effective defensive behaviour against *V. velutina* [8]. Moreover, its wide predation spectrum might also affect, more in general, insect communities and the ecosystem services they provide, such as pollination [9], although scientific evidence is currently limited. Finally, *V. velutina* represents an economic issue in the invaded countries, both for the impacts associated to the collapse of honey bee colonies [7] and the costs related to the implementation of control activities [10].

Several countries worldwide are monitoring the presence of *V. velutina*, with the aim of detecting new occurrences in areas not yet colonised by the species for the subsequent implementation of control or rapid response strategies, which are based on nest detection and destruction [4,11,12,13]. This surveillance activity is also mandatory for EU countries, since *V. velutina* is listed as an invasive alien species of Union concern (EU Regulation 1141/2016) in the framework of the respective European regulation (EU Regulation 1143/2014).

Monitoring is generally performed by integrating several approaches: (i) trapping adults, (ii) spotting nests and (iii) observing the presence of *V. velutina* on flowers or in apiaries while hunting for honey bees [4,14,15].

Several models of trap are available for trapping adults, and some of them require attractants [16]. Sugar-based baits are commonly used for attracting social wasp species [17,18] or trapping *V. velutina* queens in spring [19] or gynes in autumn, while protein-based baits are mainly used in summer for trapping workers around apiaries [16,20]. Traps with sugar-based baits have been widely used for monitoring the presence of *V. velutina* [14,21]. Furthermore, with an increased density of traps in the environment, this approach has been used as a complementary method for attempting its control by trapping queens [16,19], despite its effectiveness appears to be limited [22].

Few studies have compared the performance of traps and baits to understand the effectiveness of trapping *V. velutina* and the consequences on native insect fauna [14,19,23]. This is particularly important since the variety of available baits and traps that have been developed (with different shapes, colours and volume capacities) could lead to different attractiveness and then different results on the target species and on native ones [14,16,23]. Therefore, further investigations are required on this topic, in order to understand which combination of trap and bait could provide the best performances.

In this study, two models of traps and two sugar-based baits (beer and a commercial bait) commonly used for trapping *V. velutina* were combined to compare their effectiveness in trapping *V. velutina* and their effects on non-target insects, accordingly to the monitoring procedures generally adopted by the beekeepers. This allowed to understand which combination of trap and bait, among the tested combinations, is more effective and selective for monitoring the presence of this invasive species. Their performances were analysed in relation to the period of the year in which the sampling was performed (spring and autumn), for evaluating the presence of differences in relation to the seasonality.

## 2. Materials and Methods

### 2.1. Sites, Traps and Sampling Protocol

The sites selected for this experiment were located in Liguria (Italy), inside the area firstly invaded by *V. velutina*: site A, in the village of Sealza (Ventimiglia; N 43.80899, E 7.55111); site B, in the village of Brunetti (Camporosso; N 43.83924, E 7.60659). This area has been colonised by the species since 2013 and the density of *V. velutina* colonies increased similarly in the two sites from 2013 to 2018, ranging between 0.2 to 2.3 nests/km^2^ in site A and 0.2 to 1.9 nests/km^2^ in site B. Moreover, the two areas were similar in terms of elevation (~300 m a.s.l.) and land cover, with a predominant presence of woodlands (61% in site A and 51% in site B) together with rural and agricultural landscapes (33% in site A and 42% in site B). In addition, the distance between the two sampling sites was more than 5 km, thus no effect of their proximity was expected on the trapping results.

**Figure 1:**
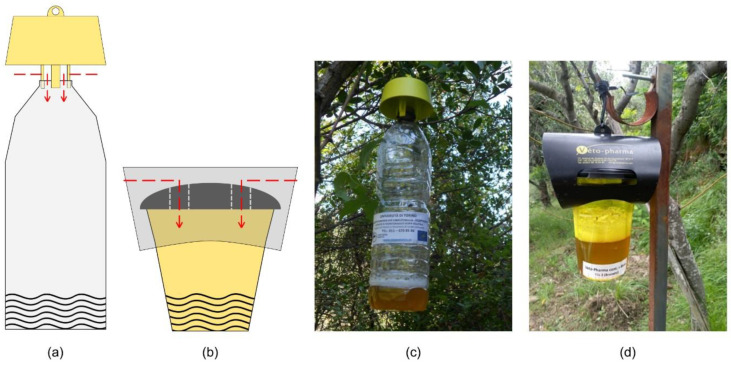
Traps tested in the study: (**a**) Scheme of the bottle trap equipped with the yellow cap (T); (**b**) scheme of the VespaCatch trap (V); (**c**) photo of the bottle trap with the yellow cap (T); (**d**) photo of the VespaCatch trap (V). The red arrows in the two schemes highlight the access route used by the hornets for entering the traps. Both models of traps were filled with beer (B) as bait (trap–bait combination named respectively TB and VB) or VespaCatch (V) attractant (trap–bait combination named respectively TV and VV) for a full factorial experiment.

In both sites, two typologies of traps (common PET bottle trap equipped with the TapTrap^®^ yellow cap; VespaCatch^®^ trap made by Véto-pharma) and two baits (common golden ale at 5% of alcohol; VespaCatch^®^ attractant made by Véto-pharma) were combined in a full factorial design and tested for evaluating their effectiveness in trapping *V. velutina* and their impacts towards non-target species (Figure 1). The two traps differed in terms of structure, colour and in the number of individual entering points, which was one for the bottle trap with the yellow cap (Figure 1a) and two for the VespaCatch trap (Figure 1b). Four trap–bait combinations were tested: TB, bottle trap with beer as bait; TV, bottle trap with VespaCatch attractant as bait; VB, VespaCatch trap with beer as bait; VV, VespaCatch trap with VespaCatch attractant as bait. Traps were filled with the same volume of bait (~250 mL) whether it was beer or the commercial attractant.

In each sampling site, 12 monitoring traps (three for each trap–bait combination) were positioned on poles at a height of 1.5 m from the ground, with a distance of 3 m between each trap and clustered per trap–bait combination. To avoid any interference between the surrounding environment and their position, each cluster was moved by one line position during each control (see Figure S1 for more details on the sampling design in each site).

Monitoring traps were activated during two seasons of 2018: In spring, from the beginning of April to the end of June (82 trapping days in site A and 84 in site B); in autumn, from the beginning of October to the end of December (70 trapping days in site A and 71 in site B). Overall, monitoring traps were checked every 25.6 ± 7.1 days; at the same time, the baits were renewed and trapped insects collected and preserved in alcohol (70%) for the subsequent taxonomic identification. The sampling interval was higher than previous studies [14,15,23] in terms of number of days between checks, since we were interested in understanding their performance in relation to the procedures adopted by the beekeepers for trapping *V. velutina*, in which checks and change of the attractive baits rarely occur on a daily or weekly basis, but customarily when apiaries are inspected and usually at intervals of 20–30 days.

### 2.2. Data Analyses: Effectiveness in Trapping V. velutina

The effectiveness of each trap–bait combination in trapping *V. velutina* during the two seasons has been evaluated with a zero-inflated GLMM model with a Poisson distribution (package *glmmTMB*). The trapped number of *V. velutina* was included as response variable, while trap model, bait typology and the season as explanatory variables, taking also into account the interactions among predictors. The sampling sites and the trapping days were included as random effects in the model. After verifying the assumptions (package *DHARMa*), the GLMM model was tested against a null-model and effects of each trap–bait combination extracted for both seasons (packages *emmeans* and *multcomp*).

### 2.3. Data Analyses: Bycatch of Insects and Differences between Trap-Bait Combinations

For both seasons, the proportion of trapped insects per taxonomic group was evaluated with a scaled PCA analysis for a first assessment of the differences between each trap–bait combination. Results of the PCA are displayed with a biplot of individuals and variables, with trap–bait combinations as grouping variable (package *factoextra*). Convex hull polygons are used for highlighting individuals from the same group of traps.

Furthermore, for each taxonomic group determined at least at the order level, the difference in the number of trapped insects per trap–bait combinations was evaluated with the Fisher’s exact test. Differences are displayed with an extended mosaic plot with standardised residuals, where cells representing negative residuals are drawn with broken borders and positive ones are drawn in solid borders, while shades of red and blue indicates different levels of standardised residuals.

### 2.4. Data Analyses: Selectiveness of the Traps

The Fisher’s exact test was used to evaluate the selectiveness of each trap–bait combination within a season, by analysing the number of non-target insects against the number of *V. velutina* trapped (2 × 4 contingency tables). A pairwise test of independence with Bonferroni correction has been applied to understand differences between groups within a season (package *rcompanion*). Results of this analysis are displayed in Table 3 as the ratio non-target insects: *V. velutina*. A value lesser than one indicates that a higher number of *V. velutina* is trapped than non-target insects, with a minimum of zero indicating no bycatches towards the non-target group; a value higher than one indicates a lower selectiveness, since a higher number of non-target insects are trapped than *V. velutina*.

All data analyses have been performed with the software R 4.0.0 [24].

## 3. Results

**Figure 2:**
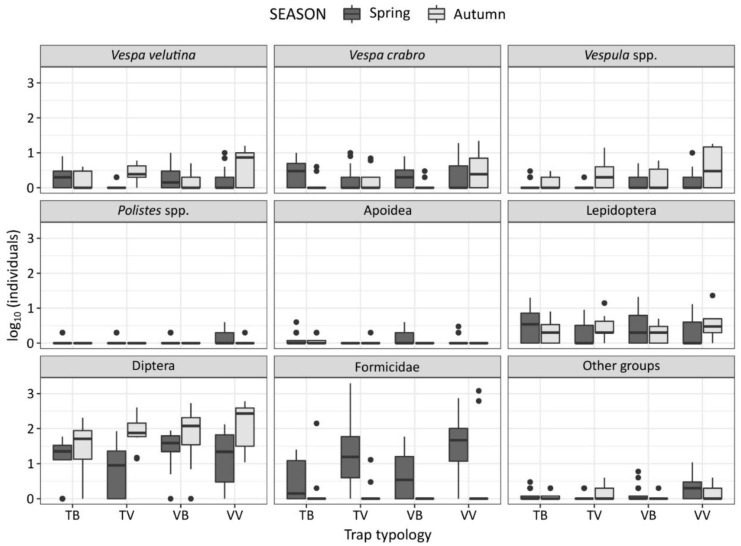
Number of individuals (log_10_ transformed) trapped in spring (dark grey) and autumn (light grey) per species or taxonomic group, and divided among trap–bait combinations (TB: bottle trap with beer; TV: bottle trap with VespaCatch attractant; VB: VespaCatch trap with beer; VV: VespaCatch trap with VespaCatch attractant).

Overall, 213 individuals of *V. velutina* have been trapped in the two sampling seasons (104 in spring and 109 in autumn), accounting for 1.02% of the total trapped insects (Table 1 and Figure 2).

A similar percentage has been recorded for *V. crabro* (261 individuals, 1.25% of the total trapped insects), but with more seasonal variation (192 individuals in spring and 69 in autumn). On the contrary, *Vespula* spp. (0.84%) and *Polistes* spp. (0.10%) were trapped with less frequency than the other wasp species. Diptera and Formicidae were the non-target groups mostly trapped either in spring or in autumn, with variations among seasons and trap–bait combinations. Apoidea (*A. mellifera*, *Bombus* spp. and other Apoidea) were not trapped frequently and, overall, they only accounted for 0.13% of the total trapped insects, while Lepidoptera catches were more frequent than those of Apoidea (382 individuals, 1.84%), but with variations among seasons and trap–bait combinations. Other groups were trapped sporadically and, overall, they accounted for 0.43% of all trapped insects.

### 3.1. Effectiveness in Trapping V. velutina

**Figure 3:**
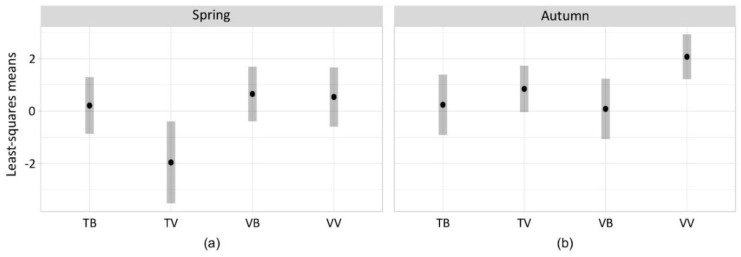
Least-squares means of *V. velutina* catches (log scale) in spring (**a**) and autumn (**b**) for each combination of trap and bait, derived from the zero-inflated GLMM of Table 2.

The variable that significantly affected the effectiveness in trapping *V. velutina* was the bait and its interaction with the model of trap and with the season, while no effect was associated to the season on its own (Table 2). In spring, traps equipped with beer as bait were more effective in trapping *V. velutina* independently from the model of trap (Figure 3; TB: LSmean = 0.21, SE = 0.55; VB: LSmean = 0.66, SE = 0.53). On the contrary, VespaCatch attractant became as effective as beer in spring only with its respective trap model (TV: LSmean = −1.96, SE = 0.79; VV: LSmean = 0.54, SE = 0.57). In autumn, the effectiveness towards *V. velutina* of traps equipped with beer decreased as well as VespaCatch attractant in bottle traps equipped with TapTrap (TB: LSmean = 0.24, SE = 0.58; VB: LSmean = 0.09, SE = 0.58; TV: LSmean = 0.85, SE = 0.45). High trapping effectiveness in autumn was maintained only by the combination of VespaCatch attractant and its respective trap model (VV: LSmean = 2.08, SE = 0.43).

### 3.2. Bycatch of Insects and Differences between Trap-Bait Combinations

**Figure 4:**
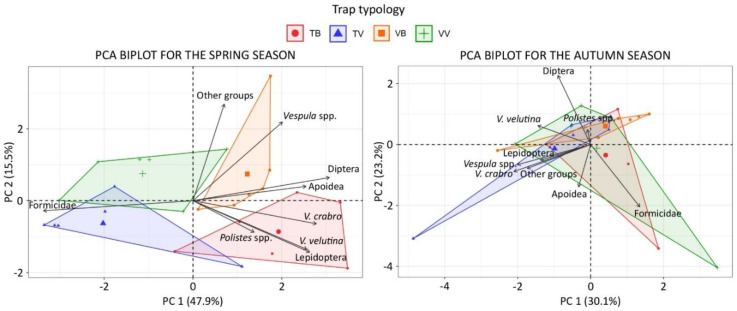
PCA biplot of individuals and variables for the spring (**left**) and autumn (**right**) season. Convex hull polygons highlight individuals of each trap–bait combination.

The PCA analysis highlighted variations in the proportion of trapped insects among trap–bait combinations in particular during the spring seasons (Figure 4). Contribution of the variables to the principal components and loading plots are reported as supplementary material (Table S1 and Figure S2).

**Figure 5:**
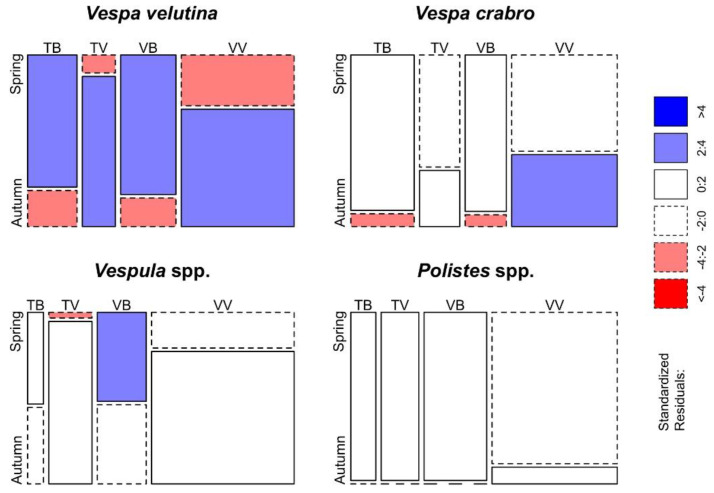
Mosaic plot of the differences between trap–bait combinations per season for the *Vespidae* family. Cells representing negative residuals are drawn in shades of red and with broken borders while positive ones are drawn in blue with solid borders.

**Figure 6:**
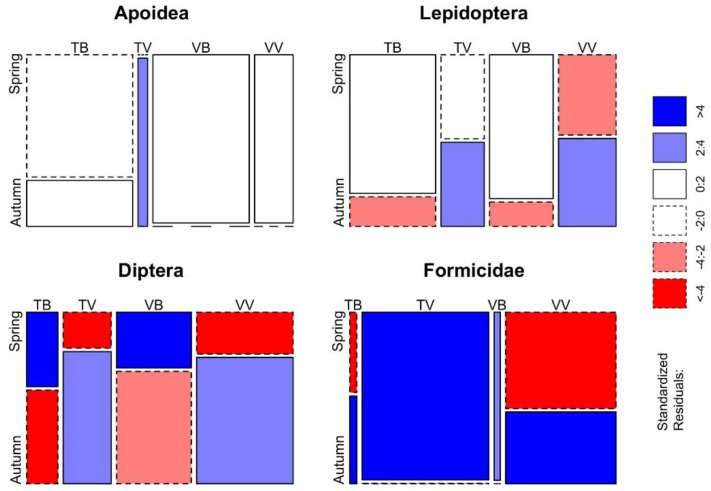
Mosaic plot of the differences between trap–bait combinations per season for Apoidea, Lepidoptera, Diptera and Formicidae. Cells representing negative residuals are drawn in shades of red and with broken borders while positive ones are drawn in blue with solid borders.

In relation to the season, the Fisher’s exact test highlighted differences between trap–bait combinations in almost all the taxonomic groups considered (*p* < 0.001 in *V. velutina*, *V. crabro*, *Vespula* spp., Lepidoptera, Diptera and Formicidae; *p* < 0.05 in Apoidea), and the only taxon without significant differences is *Polistes* spp. Differences between trap–bait combinations in the Vespidae family and in other taxonomic groups (determined at least at the order level) are displayed respectively in Figure 5 and Figure 6.

### 3.3. Selectiveness of the Traps

Overall, traps equipped with beer as bait were significantly more selective in spring than traps with the commercial bait (Table 3). In relation to *V. velutina* catches, TB and VB trapped a significant lesser number of *V. crabro*, Diptera, Formicidae and other taxa than the other trap–bait combinations. Lepidoptera were trapped frequently in spring, however VB and VV traps ensured the best ratio between bycatches of Lepidoptera and *V. velutina* catches. Concerning the other non-target groups, all trap–bait combinations trapped indifferently a small number of Apoidea, *Vespula* spp. and *Polistes* spp.

In autumn, the selectiveness of beer as bait decreases while the one of the commercial bait slightly increases (Table 3), in particular when it is associated with the commercial model of trap. In fact, VV trap allowed to significantly catch a lesser number of Diptera, Lepidoptera and Apoidea in respect to *V. velutina* catches, though Apoidea catches were infrequent with all trap–bait combinations.

## 4. Discussion

With this experiment, we compared the effectiveness and the selectiveness of two typologies of traps and two sugar-based baits that are commonly used for trapping *V. velutina* in several countries worldwide, either for monitoring or control purposes. A difference has been demonstrated among trap–bait combinations in relation to the period of the year. In spring, traps equipped with common beer as bait were trapping a higher number of *V. velutina* independently of the model of trap, while traps with a commercial attractant (VespaCatch) were effective only with its respective trap model, which therefore foresee a higher equipment cost. Conversely, the effectiveness in trapping *V. velutina* of beer-based traps decreased in autumn, and only the VespaCatch trap and bait combination maintained a higher effectiveness towards *V. velutina*. The decrease of the effectiveness of beer-based baits during the autumn might be associated to different environmental temperatures that could modify the olfactive profile of the bait.

Despite the differences associated to traps and baits, the season on its own was not affecting *V. velutina* catches, and this underlines the low performance of these traps for controlling purposes during the autumn. Since the population of *V. velutina* colonies increases along the year, with production peaks of individuals that approximately occurs during the months of October–November [25], an increase in the number of hornets in the environment is expected in autumn. However, this increase did not occurred in the number of *V. velutina* trapped during this season, and this casts doubt on the effectiveness of these traps for controlling the species in autumn.

In analogy with previous studies [19,21,23], overall *V. velutina* catches represented 1.02% of the total trapped insects, suggesting that all trap–bait combinations will generate an important impact on native insects, especially if used at high densities for controlling purposes. However, an appropriate selection of both trap model and attractant, in relation to the period of the year, could slightly increase the traps performance. This is particularly important in the framework of a monitoring strategy in which a low trap density should still be used as a complementary method for early detecting the presence of *V. velutina* in new areas and thus for the implementation of control/eradication strategies based on early nest detection and destruction [4,13]. For example, TB and VB traps in spring were either slightly more effective towards *V. velutina* (respectively 3.65% and 2.72%) and, at the same time, selective for several other groups in relation to *V. velutina* catches; notwithstanding a higher number of Diptera and Lepidoptera are trapped than those caught with traps equipped with the commercial bait, to which is however associated a significant attractiveness towards Formicidae. In autumn, VV traps performed better in terms of effectiveness towards *V. velutina*, however this trap also caught a relatively higher number of Diptera, Lepidoptera, Formicidae and *V. crabro*. Therefore, a local assessment of vulnerable species (e.g., red listed species) before the implementation of monitoring strategies may be useful for evaluating the distribution of traps in the environment, with the aim of minimising the bycatches effect of species already threatened by other external factors. Interestingly, overall Apoidea catches were quite restrained (0.09% for *A. mellifera*, 0.03% for *Bombus* spp. and 0.01% for other Apoidea), indicating a low or negligible impact on this group, which is one of the main taxa responsible for pollination. Therefore, future research for improving trapping performances should be mainly directed to minimise bycatches of Diptera, Lepidoptera, Formicidae and *V. crabro*.

Nevertheless, as previously suggested [19], the rough number of non-target insects is not sufficient for recognising negative effects on the population dynamics. For example, it could be relevant to understand, among the mostly trapped non-target groups (i.e., Diptera and Formicidae) or among a potential vulnerable group (Lepidoptera), which species have been trapped, their conservation status and the proportion of trapped insects compared to the size of the population. One example that highlight this necessity is the fact that many Diptera and Formicidae that were trapped in the two study sites are exotic species, such as the spotted wing drosophila (*Drosophila suzukii*) and the Argentine ant (*Linepithema humile*). In this case, bycatch of exotic species is not a negative result for biodiversity conservation. This may represent a complementary aspect that should be taken into account when planning future experiments on trap performances.

Another factor that could explain the high proportion of Diptera is the length of the sampling interval among checks and bait substitution. This interval has been selected to reflect the approaches generally adopted by the beekeepers for monitoring *V. velutina*, and thus understand the effects of trapping in the framework of the current procedures for monitoring the species. As the days increase, the number of dead insects in the trap increases as well while their conservation status decrease, thus this factor may attract a higher number of Diptera. However, even with the sampling interval adopted, the mean values per trap of Diptera were considerably smaller than values from other studies (528.67 ± 578.67 individuals) that adopted a shorter sampling interval of 14–15 days and similar traps [23]. Therefore, even with a longer sampling interval, the tested traps may have performed better in terms of selectiveness towards Diptera, despite this difference could also be related to different environmental and climatic conditions between the study areas (in this case Spain and Italy). This highlights the necessity to test, with a common protocol, the performance of baits and traps in all of the countries where *V. velutina* should be monitored, since results may change due to local characteristics.

In any case, the development of more selective traps and attractive compounds may provide an alternative for monitoring *V. velutina* or controlling the species with a less bycatch effect. An alternative to sugary-based traps is represented by pheromone traps, which have proved to be effective for monitoring and controlling several insect species [26,27,28]. Recently, a pheromone for attracting males of *V. velutina* has been discovered [29]. This compound could potentially find an application in autumn for monitoring the presence of males or, at a rather higher trap density, also for mating disruption strategies [1]. On the contrary, compounds associated to honey bee colonies (pollen and honey) and the honey bee aggregation pheromone have proved to be attractive for *V. velutina* workers [30]. These compounds may represent an effective alternative for monitoring or control purposes since the emerging of queens in spring and their performances should be tested against other attractive substances, such as pheromones or the baits tested in this study.

Some of the parameters that should be taken into consideration when planning future studies on trap performances are: trap model, with a focus on design, colour, number and dimension of trap’s entrance, number and dimension of escaping holes for avoiding bycatches of smaller species; bait typology, volume and changes in its composition in relation to the sampling interval; period of the year; sampling intervals between bait’s replacement; *V. velutina* density; local characteristics (e.g., climatic conditions, land-use) surrounding the experiment. Testing these parameters in several countries and several areas within each country, with common protocols, and with the possibility to identify trapped insects at the species level, including other taxa rather than Hymenoptera, would contribute to improve trap performances.

## 5. Conclusions

This experiment provides information on the effectiveness of sugary-based traps for catching the invasive hornet *V. velutina* and their potential effects on non-target insects, taking into account the seasons (spring and autumn) in which sugary-based traps are mostly used. Differences between traps and baits were demonstrated: in spring, traps equipped with common beer as bait were both more effective and selective; in autumn, higher performance in terms of both effectiveness and selectiveness were obtained by just one of the four trap–bait combinations. Bycatches of Apoidea were negligible, while other groups were more represented, thus further selective trapping methods should be investigated in the future, particularly when traps are used at a high density for control rather than for monitoring purposes.

## Figures and Tables

**Table 1:** Overall trapping results in the two sampling sites (total column) and results divided between spring and autumn taking into account the four trap–bait combinations. For each period, the mean number of individuals per trap (standard deviation in brackets) and the percentage of trapped individuals for the corresponding species/group out of the total number of trapped insects are reported.

	TOTAL			SPRING								AUTUMN							
				TB		TV		VB		VV		TB		TV		VB		VV	
	N. of Individuals	Mean per Trap (*SD*)	% of Insects	Mean per Trap (*SD*)	% of Insects	Mean per Trap (*SD*)	% of Insects	Mean per Trap (*SD*)	% of Insects	Mean per Trap (*SD*)	% of Insects	Mean per Trap (*SD*)	% of Insects	Mean per Trap (*SD*)	% of Insects	Mean per Trap (*SD*)	% of Insects	Mean per Trap (*SD*)	% of Insects
Hymenoptera																			
Vespoidea																			
*Vespa velutina*	213	1.48 (2.64)	1.02	1.38 (1.78)	3.65	0.13 (0.33)	0.06	1.63 (2.36)	2.72	1.21 (2.36)	0.80	0.75 (1.09)	1.02	2.08 (1.71)	1.53	0.67 (1.11)	0.39	5.58 (5.22)	1.35
*Vespa crabro*	261	1.81 (3.16)	1.25	2.54 (2.74)	6.74	1.17 (2.36)	0.52	1.67 (2.25)	2.79	2.63 (4.09)	1.74	0.42 (0.95)	0.57	1.17 (1.99)	0.86	0.25 (0.60)	0.15	3.92 (5.72)	0.95
*Vespula* spp.	174	1.21 (3.08)	0.84	0.25 (0.52)	0.66	0.04 (0.20)	0.02	0.75 (1.13)	1.26	0.88 (1.92)	0.58	0.42 (0.64)	0.57	2.42 (3.64)	1.78	1.33 (1.84)	0.79	6.50 (7.16)	1.57
*Polistes* spp.	20	0.14 (0.40)	0.10	0.08 (0.28)	0.22	0.13 (0.33)	0.06	0.21 (0.41)	0.35	0.38 (0.70)	0.25	0.00 (0.00)	0.00	0.00 (0.00)	0.00	0.00 (0.00)	0.00	0.08 (0.28)	0.02
Formicidae	9641	67.0 (258.5)	46.35	5.4 (7.7)	14.26	202.8 (518.8)	90.95	9.9 (14.5)	16.55	101.6 (173.8)	67.50	11.8 (38.7)	15.99	1.2 (3.3)	0.86	0.0 (0.0)	0.00	151.3 (358.7)	36.63
Hymenoptera Apoidea																			
*Apis mellifera*	18	0.13 (0.45)	0.09	0.29 (0.68)	0.77	0.00 (0.00)	0.00	0.33 (0.75)	0.56	0.08 (0.28)	0.06	0.08 (0.28)	0.11	0.00 (0.00)	0.00	0.00 (0.00)	0.00	0.00 (0.00)	0.00
*Bombus* spp.	6	0.04 (0.20)	0.03	0.04 (0.20)	0.11	0.00 (0.00)	0.00	0.08 (0.28)	0.14	0.08 (0.28)	0.06	0.00 (0.00)	0.00	0.08 (0.28)	0.06	0.00 (0.00)	0.00	0.00 (0.00)	0.00
Other Apoidea	2	0.01 (0.12)	0.01	0.00 (0.00)	0.00	0.00 (0.00)	0.00	0.00 (0.00)	0.00	0.00 (0.00)	0.00	0.17 (0.37)	0.23	0.00 (0.00)	0.00	0.00 (0.00)	0.00	0.00 (0.00)	0.00
Diptera	9993	69.4 (111.3)	48.04	23.0 (16.5)	60.89	17.1 (23.6)	7.68	41.2 (27.1)	68.99	39.9 (45.3)	26.52	57.8 (56.7)	78.56	125.7 (117.2)	92.52	165.9 (170.0)	97.93	241.1 (200.8)	58.39
Lepidoptera	382	2.65 (3.97)	1.84	4.50 (5.09)	11.93	1.38 (2.29)	0.62	3.46 (4.68)	5.80	1.75 (2.74)	1.16	1.92 (2.36)	2.61	2.75 (3.39)	2.02	1.17 (1.28)	0.69	3.83 (5.62)	0.93
Other groups	90	0.63 (1.49)	0.43	0.29 (0.54)	0.77	0.21 (0.41)	0.09	0.50 (1.15)	0.84	2.00 (2.86)	1.33	0.25 (0.43)	0.34	0.50 (0.87)	0.37	0.08 (0.28)	0.05	0.67 (0.94)	0.16

**Table 2:** Conditional model of the zero-inflated GLMM for evaluating the effectiveness in trapping *V. velutina*. The effect size of the predictors (*β*), the corresponding standard error (*SE*), *Z* and *p* values are reported. The footnotes describe the structure of the model and the result of the comparison against a null-model.

Variables	*β*	*SE*	*Z*	*p*
Season (Autumn)	0.03	0.71	0.04	0.968
Bait (VespaCatch)	−2.18	0.63	−3.44	<0.001
Trap_model (VespaCatch)	0.44	0.26	1.71	0.088
Season (Autumn): Bait (VespaCatch)	2.78	0.79	3.52	<0.001
Season (Autumn): Trap_model (VespaCatch)	−0.60	0.64	−0.94	0.348
Bait (VespaCatch): Trap typology (VespaCatch)	2.06	0.69	2.97	<0.01
Season (Autumn): Bait (VespaCatch): Trap typology (VespaCatch)	−0.68	0.95	−0.71	0.475

GLMM Model: *Vespa* *velutina* ~ Bait typology * Trap typology * Season + (1|Study area) + (1|Trapping days). Comparison between the selected model (AIC 436) and the null-model (AIC 521): *χ^2^* = 101, *df* = 8, *p* < 0.001.

**Table 3:** Results of the Fisher’s exact test and of the pairwise test of independence with Bonferroni correction for the analysis of the selectiveness. For each comparison, *p*-values of the Fisher’s exact test are reported. Values for each trap–bait combination represent the ratio non-target insects: *V. velutina*. Letters define similarities among groups in agreement with a pairwise test of independence with Bonferroni correction; letters are ordered alphabetically in relation to their degree of selectiveness (a * = higher degree of selectiveness than the other trap–bait combination).

	SPRING									AUTUMN								
	*p*	TB		TV		VB		VV		*p*	TB		TV		VB		VV	
Apoidea	0.763	0.24	a	0.00	a	0.26	a	0.14	a	<0.01	0.33	b	0.04	ab	0.00	ab	0.00	a *
*Vespa crabro*	<0.001	1.85	a *	9.33	b	1.03	a *	2.17	ab	0.798	0.56	a	0.56	a	0.38	a	0.70	a
*Vespula* spp.	0.047	0.18	a	0.33	a	0.46	a	0.72	a	0.339	0.56	a	1.16	a	2.00	a	1.16	a
*Polistes* spp.	0.017	0.06	a	1.00	a	0.13	a	0.31	a	1.000	0.00	a	0.00	a	0.00	a	0.01	a
Formicidae	<0.001	3.91	a *	1622	c	6.08	a *	84.10	b	<0.001	15.67	b	0.56	a *	0.00	a *	27.09	b
Diptera	<0.001	16.7	a *	137	b	25.33	a *	33.03	ab	<0.001	77.00	ab	60.32	a *	248	b	43.18	a *
Lepidoptera	<0.01	3.27	ab	11.00	b	2.13	a *	1.45	a *	<0.05	2.56	b	1.32	ab	1.75	ab	0.69	a *
Other groups	<0.001	0.21	a *	1.67	ab	0.31	a *	1.66	b	0.365	0.33	a	0.24	a	0.13	a	0.12	a

* Trap-bait combinations that catch a lower number of non-target insects in proportion to *V. velutina* catches.

## Supplementary Materials

The following are available online at https://www.mdpi.com/2075-4450/11/10/706/s1, Figure S1: Trapping structure of each sampling site. Figure S2: Loading plots of the first and second components of the PCA analysis on spring and autumn data. Table S1: Contribution of the variables to the first, second and third components of the PCA analysis on spring and autumn data. Table S2: Experimental data.
