# Supplementary material for: Effectiveness and Selectiveness of Traps and Baits for Catching the Invasive Hornet Vespa velutina
Source: Insects. 2020 Oct 16;11(10):706. doi: 10.3390/insects11100706 (PMC7602873; doi:10.3390/insects11100706)
Supplement: Supplementary file 1 [file insects-11-00706-s001.zip › SUPPLEMENTARY MATERIALS/Traps and baits for trapping Vespa velutina - Fig S1, Fig S2 & Table S1.docx]

Effectiveness and selectiveness of traps and baits for catching the invasive hornet *Vespa velutina*

**Simone Lioy, Daniela Laurino, Michela Capello, Andrea Romano, Aulo Manino and Marco Porporato**

**Supplementary Materials**

**Figure S1**. Trapping structure of each sampling site: TB, bottle trap with TapTrap and common beer as bait; VB, VespaCatch trap and common beer as bait; TV, bottle trap with TapTrap and VespaCatch attractant as bait; VV, VespaCatch trap and VespaCatch attractant as bait. At every check, baits were renewed and trap clusters shifted of one line position.


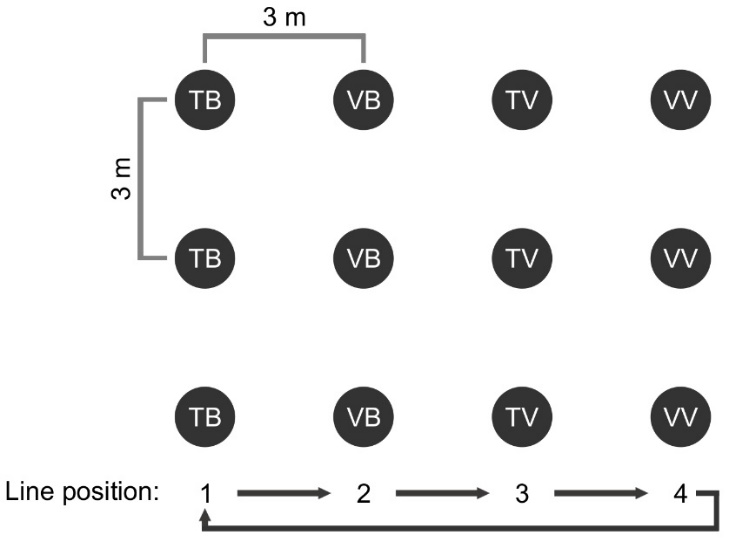


**Figure S2**. Loading plots of the first and second components of the PCA analysis on spring (a) and autumn data (b).


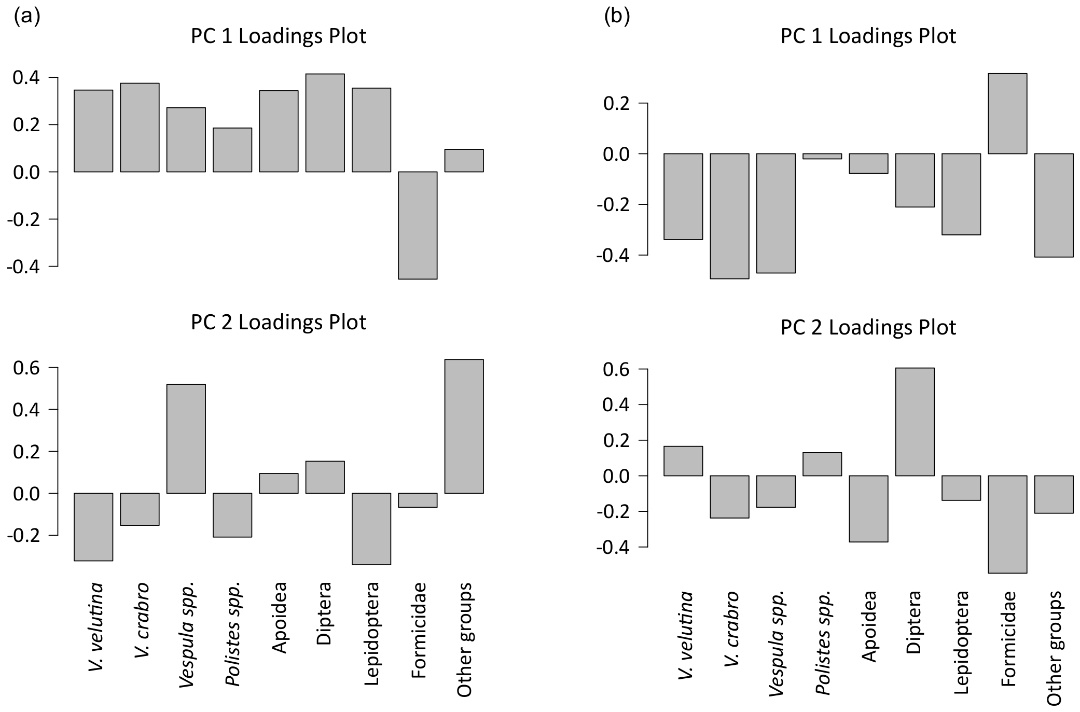


**Table S1**. Contribution of the variables to the first, second and third components of the PCA analysis on spring and autumn data.

| **Season** | **Species/Group** | **PC1** | **PC2** | **PC3** |
| --- | --- | --- | --- | --- |
| **Spring** | *Vespa velutina* | 11.99 | 10.36 | 0.39 |
|  | *Vespa crabro* | 14.08 | 2.33 | 7.92 |
|  | *Vespula* spp. | 7.39 | 27.03 | 0.62 |
|  | *Polistes* spp. | 3.45 | 4.35 | 79.14 |
|  | Apoidea | 11.80 | 0.89 | 2.30 |
|  | Diptera | 17.19 | 2.34 | 0.88 |
|  | Lepidoptera | 12.58 | 11.58 | 8.58 |
|  | Formicidae | 20.63 | 0.45 | 0.06 |
|  | Other groups | 0.90 | 40.68 | 0.10 |
| **Autumn** | *Vespa velutina* | 11.47 | 2.74 | 25.00 |
|  | *Vespa crabro* | 24.36 | 5.63 | 1.28 |
|  | *Vespula* spp. | 22.18 | 3.14 | 0.06 |
|  | *Polistes* spp*.* | 0.04 | 1.69 | 23.40 |
|  | Apoidea | 0.60 | 13.84 | 15.40 |
|  | Diptera | 4.43 | 36.67 | 6.25 |
|  | Lepidoptera | 10.25 | 1.90 | 18.70 |
|  | Formicidae | 10.02 | 29.97 | 6.30 |
|  | Other groups | 16.64 | 4.43 | 3.61 |

**Table S2**. Experimental data (see the attached .xlsx file).
